# Inflectional zero morphology – Linguistic myth or neurocognitive reality?

**DOI:** 10.3389/fpsyg.2022.1015435

**Published:** 2022-12-08

**Authors:** Maria Alekseeva, Andriy Myachykov, Yury Shtyrov

**Affiliations:** ^1^Centre for Cognition and Decision Making, Higher School of Economics, Institute for Cognitive Neuroscience, Moscow, Russia; ^2^Department of Psychology, Northumbria University, Newcastle upon Tyne, United Kingdom; ^3^Center of Functionally Integrative Neuroscience (CFIN), Department of Clinical Medicine, Aarhus University, Aarhus, Denmark

**Keywords:** linguistic theories, universal grammar, null constituent, zero morpheme, inflectional zeroes

## Abstract

Knowledge of language, its structure and grammar are an essential part of our education and daily activities. Despite the importance of language in our lives, linguistic theories that explain how the language system operates are often disconnected from our knowledge of the brain’s neurocognitive mechanisms underpinning the linguistic function. This is reflected, for example, in the inclusion of abstract and often controversial elements into theories of language. Here, we discuss the case of the so-called *null constituent* and its smallest and the most controversial variant – the *zero morpheme*, a hypothetical morphosyntactic device that has no overt physical (phonological or orthographic) expression. Focusing on the putative inflectional zero morpheme, we discuss the theoretical origins and pitfalls of this approach and advocate the important role for neurobiological research that could try to elucidate the neurocognitive reality of such constructs in linguistic communication.

## Introduction

### On the “zero element” term

The phenomenon of a defunct object was addressed in classical philosophy, where it was seen as contradicting itself (Hume, 1896; Smith, 2011). The problem directly related to the notion of zero units in phenomenology is grounded in the paradox of impossibility to produce a true statement of a non-existing object, whereas the retrieval and production process implies the objective existence of such an object. This paradox features in different fields, often far beyond abstract phenomenological considerations, naturally extending to the study of language and its structural units.

Language is one of the most sophisticated products of human evolution that supports communication and thus enables all our individual, social, economic, cultural, and intellectual activities. However, conventional linguistic theories do not always take into account the knowledge of the neurobiological foundations of the language function in the human brain. As a result, they often rely upon highly abstract and derived models that include categories and notions not necessarily represented overtly in linguistic behavior and enjoy little-to-no support from experimental research. One such example is the so-called *zero morpheme.* It is posited that zero morpheme is a morphosyntactic unit without overt graphemic and phonological counterparts: e.g., compare “*cat****-Ø***” (singular) vs. “*cat****-s***” (plural) where the singular inflectional form is allegedly represented only implicitly while the plural form receives the explicit *-s* marker. This “invisible” member of the corresponding category (conventionally marked with the zero sign “Ø” in linguistics) is purported to be necessary for efficient linguistic processing, and is assumed to be a part of most languages’ grammars. Since its representation is not accompanied by any physical referent, there is a widespread controversy surrounding the nature and the neurocognitive reality of zero morphology – in relation to both individual languages and the universalist approaches to grammar.

This problem is inherently paradoxical – even from the viewpoint of its accessibility for empirical investigation as it makes experimental hypothesis falsification somewhat problematic (Popper, 1972). As mentioned above, zero morpheme reality is already included in the language grammars, making it impossible to falsify (or operationalize the conditions demonstrating its non-existence) due to its incommensurability (Kuhn, 1962). In this brief overview, we discuss some debatable issues related to zero morphology in the context of its neurocognitive reality and suggest experimental approaches that should help circumvent the falsification issue and better inform linguistic theories.

One of the founding fathers of the 20th-century linguistics, Ferdinand de Saussure, defined language as a system where the absence of overt features is as important as their presence (Saussure, 1916). This characterization contributed to the development of the idea of a null constituent defined later by Bally (1932) as a sign that has a particular meaning but is not embodied in sounds; in other words, zero constituents are units that carry out a grammatical or a semantic function without having any overt phonological or graphemic representations. Since then, several null constituents have been identified by theorists: ellipsis, wh-movements, NP-movements, and word-bound morphological elements – the so called zero morphemes (see more: ellipsis – Merchant, 2019; wh-movements – Chomsky, 1977; NP-movements – Chomsky, 1981; zero morphemes – Dahl and Fábregas, 2018).

### Zero morpheme and its theoretical implications

Being placed within single words, zero morphemes stand out among other null constituents and are understood as covert markers – inflectional or derivational. For example, the Universal Grammar (UG; Chomsky, 1965) proposed both overt and zero inflectional affixes in Slavic case marking [e.g., singular masculine noun “*stol*” (table) in Russian nominative and dative cases: “*stol-***Ø***_*Nom*_” – “stol-****a****_*Dat*_”*] for the purposes of saving the structural system in universal templates. Thus, we have an element without the phonological level of representation, which is, however, attributed with a morphological representation and an abstract internal syntactic function. According to the UG, which postulates a relatively universal structure of all language systems that can be expressed in a specific set of rules, the human brain contains a limited set of constructs for organizing the language system, thus necessitating zero morphemes where overt “classical” ones are not obviously present. By contrast, the Lexical Morphology hypothesis (LMH), derived from Baudouin de Courtenay’s (1895) and Bloomfield’s (1933) framework, postulates that all lexemes and morphological units should be fully specified phonologically, following the “classical” morpheme idea. Accordingly, Aronoff (1994) suggested to treat zero inflection and derivation as a lexeme formation without a morphological representation thus rendering zeroes outside of morphology units. Further, LMH was followed by a newer morphological theory, which relatively successfully explained the zero-morpheme problem by stepping away from the traditional idea of morphological units – Lexeme-Morpheme Base Morphology (LMBM; Beard, 1995). It still postulates the existence of phonologically overt lexemes (direct articulation of meaning by sound), while grammatical morphemes are defined as an indirect means of reference (conditioned indirect articulation).

At the same time, Anderson (1992) proposed the so-called A-Morphous Morphology theory that includes impoverished morphological processes arguing for an independence of syntactic features from phonological representations and even proposing to eliminate affixes from morphology. Anderson (1992) also suggested that syntactic features like inflections are heads of the functional categories and therefore can be used as separate terminal nodes (separate morphemes). In the case of empty phonological representations of such nodes, the feature can be added to the one with an overt representation, thus having several features in one morpheme and offering another possible solution to the “zero morpheme” question.

Finally, Distributed Morphology approach (Halle and Marantz, 1993; Oltra-Massuet, 1999), combined the approaches of lexical and A-Morphous Morphology theories. To address the zero-morpheme question as a morphological gap, it proposes a binary nature (value) of different features in our mental lexicon. When a positive value of the feature is deleted, the negative value is attributed automatically, i.e., it becomes the default one, thus forming the zero representation. A similar approach is taken by the Minimalist Morphology where zero affixes are not zeroes as such but are examples of a negative feature value (Wunderlich and Fabri, 1995).

### Experimental questions

Cases of putative zero morphology can be found in both inflection (case, tense, number, person, and aspect marking, e.g*., “I walk-Ø” – “she walk-s”*) and derivation (other category formation; e.g., “*to walk_*Verb*_” – “a walk-Ø_*Noun*_”*). Until now, several questions were raised regarding derivational zeroes (Myers, 1984; Pesetsky, 1995; Plag, 1999). The most problematic issue regarding zero derivation is the absence of an overt analog with a similar function (Sanders, 1988). For the inflectional zeroes, however, there is typically a binary structure where we can identify an overt counterpart (“*table-Ø” – “table-s”*). Thus, arguably the optimal way to investigate the cognitive reality of zeroes is to start from their easiest and less controversial cases – inflectional zeroes, such as those in case, gender, or number marking. While we will not consider in detail the special cases where zero inflection entails phonological changes in the root, such as in irregular verbs and noun plurals (e.g., “*mouse_*SG*_” – “mice-Ø_*PL*_,”* not “*mouse*+*s_*PL*_”*; *“to get*” – past tense “*got*-Ø”, not “*get+ed*”) due to the more complicated/multiple processes involved, we will briefly discuss some other pitfalls that beset empirical studies in this field.

Language diversity is the source of the first challenge. Inflectional zeroes in different languages are processed by native speakers in different ways. On the one hand, in English with its impoverished inflectional system, covert affixes are considered more common than overt ones. So, verbal stems, for example, can be used without any overt affixes in most cases, except past-tense “*-ed”* and 3rd person present singular “*-s*.” Nouns, in turn, use only plural “*-s*,” whereas adjectives have no overt affixation at all. On the other hand, languages with richer inflectional systems, e.g., Finno-Ugric, Germanic, Romance, or Slavic, typically require overt affixation in most cases. Thus, inflection is a peripheral feature in English while it is a core one in German or Russian. One of the possible suggestions (following the UG and other related null-constituent frameworks) is to assume the presence of zero affixes in one language but not in another. Developmental data indeed offer some support to this view: For instance, English-speaking children produce more single stem (without overt affixes) forms in contrast to their German or Russian-speaking peers who rarely produce forms lacking overt affixation (Slobin, 1973; Clahsen, 1986; Hyams, 1987; Marchman, 1997). Moreover, the “avoid zero affixation generalization” theory suggests that children avoid zero-marked forms during the initial stages of grammar learning and that they are able to add specific rules related to the use of zero forms at the later grammar-learning stages (Hyams, 1987). This system indeed can feature prominently in the languages with explicit inflection.

At the same time, studies using past-tense verb production task in English show a higher proportion of “omission errors” (otherwise zero-marking) for young children, decreasing with age (Matthews and Theakston, 2006; see also for the number of zero-inflected errors). On the other hand Marinis and Chondrogianni (2010) showed that native English-speaking children produce fewer zero-marked errors than overregularization errors (see also Marchman et al., 1999 and Conti-Ramsden et al., 2011 for experiments with adults). Moreover, Berko (1958) showed that English-speaking children made more mistakes when producing plural than singular possessive forms, suggesting the existence of default forms as proposed by the Distributed Morphology account.

While this discussion can adequately address decomposition processes where we divide wordforms into stems and affixes marking potential features, it also raises a further question regarding words’ whole-form storage vs. morphological decomposition as well as their encoding and retrieval from memory with or without morphological features (Bybee, 1995; Taft, 2004). A potential solution is suggested by the dual-system theory (Pinker, 2015). This approach suggests an early and a largely automatic access to all potential complex forms similar to overt and regularly inflected ones, which by default requires morpho-phonological parsing in order to segment complex forms into stems and affixes (e.g., “*corner”* and “*band”* are automatically segmented into “*corn*+*er”* and “*ban*+(e)*d*,” a parsing attempt later discarded; Marslen-Wilson and Tyler, 2007). At the same time, the dualistic nature of this approach includes whole-form storage for specific units. Thus, while the overt forms need to be decomposed, the zero ones can be thought of as basic forms stored as integral wholes that do not require parsing/segmentation. This approach clearly entails that there are no additional zero-morpheme representations in the brain’s neurolinguistic repository, making it impossible to separate stem morphemes from the putative zero ones – and thus undermines potential experimental attempts at testing their existence.

Indeed, despite the abundance of words with no overt affixation across languages and an extended theoretical use of zero affixes in linguistics, there is little experimental psycholinguistic or neurolinguistic evidence documenting the neurocognitive reality of zero morphemes. In the absence of an optimal neurocognitive theoretical framework, one has to proceed with the assumption that it is not possible to ensure the presence of a null element unless one could mechanistically separate syntactic (or morphosyntactic) processing traces from the semantic ones (Fodor, 1993). That is, even if we could design a morphosyntactic experiment incorporating zeroes, this could yield no conclusive evidence regarding zero constituent’s existence, but may instead only speak to a parsing process of detecting a gap in its place (Sag and Fodor, 1994). For example, in assessing the processing of an English present-tense verb “*buy”* in “*they buy-Ø_*PL*_”* (as opposed to “*he buy*+*s_*SG*_”*) or a similar Swedish noun-number marking contrast [“*Eltern-Ø_*PL*_” (parents*) vs. “*Eltern*+*teil*_*SG*_” (*parent*)], the final stage of processing needs to be disambiguated between that of the zero morpheme (number marker) *per se* simply and the detection of a “missing something” at that point (for example the overt gender or number marker that hypothetically could be present). As a result, experimental research has not yet succeeded in detecting traces of zero morpheme retrieval that could help elucidate its neurobiological and cognitive reality with any certainty. In the next section, we will suggest some possible ways for tackling this problem.

Despite the problems associated with investigating the neurocognitive mechanisms of zero morphemes, there have been already some attempts to trace zero morpheme access in linguistic behavior. For example, Vasilyeva (2018) used a visual lexical decision task in a study which documented processing advantage of Russian zero noun forms over the overtly affixed ones showing faster reaction times (RTs) for written zero nominative case than for overt oblique case masculine forms, and for zero nominative masculine forms vs. overt nominative feminine forms. These findings indicate that there is no extra processing cost for zeroes, supporting the whole-word storage for the zero-forms and thus the absence of zero affix representation in our cognitive system. On the other hand, a similar contrast between nominative and oblique cases with zero and overt inflectional affixes was tested by Gor et al. (2017) using auditory word presentation. Their results showed comparable reaction times for both overt and zero-inflected forms reinforcing the issues raised by Sag and Fodor (1994) regarding checking costs during gap processing and recombination.

At the brain level, the available evidence is even more scarce. Sahin et al. (2006) explored the role of Broca’s area in morphological processing of different inflection types – regular, irregular, and zero using a cued covert production task in a functional magnetic resonance imaging (fMRI) experiment. The study showed a significant activation of Broca’s area in zero-inflected conditions suggesting morphological processing of zero inflection morphemes and thus hinting at the existence of their neural representations. However, this explanation is not unequivocal as the presence of morphological processing in the zero affix trials does not necessarily indicate the existence of zero affix representations as such, as it may simply indicate standard parsing of the stimuli which include other morphological parts (e.g., stems) or suggest a different approach to the extraction of the zero-inflected forms (whole-word storage vs. full decomposition). Furthermore, fMRI lacks adequate temporal resolution optimal for tracking online language processing by the brain, thus potentially confounding results by late task-irrelevant post-comprehension brain activity (Kim et al., 1997).

## Suggested directions

Following the dual-system approach and considering the recent findings regarding a very early timing of zero-inflected form processing, one could try addressing the question of the neurocognitive reality of zero morpheme representations by focusing on the time-course of their putative access in the brain. This would require neuroimaging methods allowing high temporal resolution, such as magnetoencephalography (MEG) or electroencephalography (EEG) that can track neuronal activity with a millisecond precision – an essential feature for highly dynamic linguistic processes (Shtyrov and Stroganova, 2015). Importantly, because we still cannot unequivocally register access to morphemes as individual representations, experimental designs should not only directly compare zero and overt forms but also manipulate the functions of zero and overt markers by providing the context which would require the retrieval of the features encapsulated in and/or affixed to the word. This, in turn, can be achieved by adopting the already existing strategy extensively employed in mainstream EEG studies of overt morphosyntax processing (Friederici et al., 1993; Hagoort et al., 1993; Osterhout and Mobley, 1995; Coulson et al., 1998) – contrasting grammatically correct and grammatically incorrect cases of inflectional morpheme use. Compare, e.g., recording the brain’s responses to such stimulus contrasts as “*we walk-s” vs. *“he walk-s”* and “*we walk-Ø”* vs. “*he walk-Ø”, where physically the same zero and overt surface forms are rendered as correct or incorrect (*) by the context of the preceding pronoun. Thus, even though event-related potentials (ERPs) in response to both overt (*-s*) and zero (*-Ø*) affixes are elicited by the same *acoustic* events in each contrast, the *linguistic* events diverge, affecting language-specific brain activation. Using such an approach, the main research question could be addressed by implicating a *morphosyntactic priming* theory (Oltra-Massuet et al., 2017) that can in principle also be applied to the specific case of zero morpheme. According to this theory, there is a prime element during phrase processing, which pre-activates (i.e., primes) the processing of the next word’s morphosyntactic features. The registration of such priming effects during the processing of zero forms would suggest an existence of zero morpheme representations by means of morphosyntactic priming effects. For example, the Early Left-Anterior Negativity (ELAN) and syntactic Mismatch Negativity (sMMN) ERP components, typically elicited by contextually erroneous overt inflections, were suggested to reflect these morphosyntactic pre-activation processes: the stimulus morpheme elicits a smaller-amplitude response when primed by a felicitous context as opposed to when it is violated by a grammatically incorrect one (as in “*he walk-s*” vs. “*we walk-s”; Pulvermüller and Shtyrov, 2003; Shtyrov et al., 2003; Hasting et al., 2007). If a similar ELAN pattern could be registered for zero affixation (e.g., a larger response for erroneous “*he walk-Ø” over *“we walk-Ø*”), its presence (potentially along with a family of other syntax-specific neural correlates, such as LAN, P600) could lend support to zero-morpheme brain activation, demonstrating an overt ERP response to the elusive covert zero affix, thus supporting neurophysiological reality of zero’s representation in the brain.

To address this issue in a more comprehensive way, different types of verbal, adjectival, and noun inflections can be used in this type of designs. Furthermore, this approach can be extended by an additional contrast–unprimed single verb with zero inflectional marker – where we can limit the impact of the contextual effects on the selection of a particular affix (zero or overt), thus introducing an additional control condition. Moreover, such unprimed condition manipulation could be used to address the question of the default zero-forms in different parts of speech. Such an approach could be used in the future to tackle different zero-inflection morphemes (e.g., for gender, case, or number agreement) in different word classes. Furthermore, it could be applied cross-linguistically to address the question of the universality of zero constituents predicted by the UG framework and the availability/absence of zero inflections in typologically diverse languages. In addition to recording ERPs, such a design could also be used to track oscillatory activity of the brain’s networks across different bands, known to underpin morphosyntactic processing, as well as delineate their cortical spatio-temporal patterns and neuroanatomical substrates (Jensen et al., 2019).

Furthermore, this approach could be adapted to psycholinguistic experiments, implementing different paradigms based on the morphosyntactic priming theory (see Bates et al., 1996 for a paradigm example on gender priming; Dahan et al., 2000 – for an eye-tracking example). It can also help address the question of zero morphemes and their representations in different contexts. Many languages (e.g., Slavic and Finno-Ugric) include ambiguous noun cases where, for example, both nominative and accusative forms have zero markings with the nominative form as the default case. This allows a range of comparisons between different marking combinations in a priming paradigm: Both Nominative and Accusative zeroes, both overt, or mixed combinations in a priming paradigm. This will help to elucidate the question of zero morpheme reality as well as examine how zero forms manifest in both default and non-default case contexts. Finally, the morphosyntactic priming approach will allow measuring both chronometric (response times) and categorical (accuracy) signatures of processing as well as multiple eye-movement parameters (e.g., saccades and gaze/fixation durations) making it possible to address the full range of behavioral and neural indices of these enigmatic language elements.

## Conclusion

In this paper, we briefly discussed the existing evidence regarding the neurocognitive reality of one of the smallest and most evasive concepts of the language grammar, the zero morpheme, and suggested potential research avenues that will foster better understanding of the neurophysiological foundations of this phenomenon. Future research will be able to verify the existence of neurobiological and behavioral signatures of accessing such non-overt, abstract morphemes. It will also shed light on the bases of morphosyntactic processing in general. In addition, this line of research will offer an “experimental hand” to the linguistic theories of zero morphology and improve our understanding of more complex null constituents as well as other abstract theoretical constructs. This, in turn, will lead to a better dialogue between linguistic and neuroimaging research fields and advance our understanding of the nature of language faculty.

## Data availability statement

All further inquiries can be directed to the corresponding author.

## Author contributions

All authors listed have made a substantial, direct, and intellectual contribution to the work, and approved it for publication.
